# Clinical Outcome of Eradication Therapy for Gastric Mucosa-Associated Lymphoid Tissue Lymphoma according to* H. pylori* Infection Status

**DOI:** 10.1155/2016/6794848

**Published:** 2016-02-29

**Authors:** Ju Seok Kim, Sun Hyung Kang, Hee Seok Moon, Jae Kyu Sung, Hyun Yong Jeong

**Affiliations:** Division of Gastroenterology, Department of Internal Medicine, Chungnam National University School of Medicine, Daejeon 35015, Republic of Korea

## Abstract

*Background*. To evaluate the long-term outcome of* H. pylori* eradication therapy for gastric MALT lymphoma according to the presence of* H. pylori* infection.* Methods*. We retrospectively reviewed the medical records of patients between January 2001 and June 2014. The clinicopathologic characteristics and clinical outcomes were compared between* H. pylori*-positive and* H. pylori*-negative gastric MALT lymphoma groups.* Results*. Fifty-four patients were enrolled: 12* H. pylori*-negative and 42* H. pylori*-positive patients. The tumor was located more frequently in both the proximal and distal parts of the stomach (*P* = 0.001), and the percentage of multiple lesions was significantly greater in the* H. pylori*-negative group (*P* = 0.046). Forty-seven patients received initial eradication therapy, and 85% (35/41) of* H. pylori*-positive patients and 50% (3/6) of* H. pylori*-negative patients achieved complete remission after eradication therapy. The presence of multiple lesions was a predictive factor for unresponsiveness to* H. pylori* eradication (*P* = 0.024). The efficacy of eradication therapy (*P* = 0.133), complete remission (CR) maintenance period, and relapse after eradication therapy were not significantly different between the two groups.* Conclusions*.* H. pylori* eradication therapy could be an effective first-line treatment for localized* H. pylori*-negative gastric MALT lymphoma, especially for single lesions.

## 1. Introduction

Mucosa-associated lymphoid tissue (MALT) lymphoma is a type of extranodal B-cell lymphoma; of these, gastric MALT lymphoma is the most common [1]. More than 90% of gastric MALT lymphoma cases are associated with* Helicobacter pylori* (*H. pylori*) infection, and several studies since 1993 have proven the effectiveness of eradication therapy, reporting lymphoma regression after therapy completion [2, 3]. Complete remission (CR) has been reported in approximately 80% of patients with gastric MALT lymphoma after eradication therapy, with recurrence rates of approximately 5% [4]. With such favorable clinical outcomes, eradication therapy is recommended as the first-line treatment for patients positive for* H. pylori* with localized gastric MALT lymphoma [5, 6]. However, little is known about the clinicopathologic characteristics and clinical course according to the* H. pylori* infection status in gastric MALT lymphoma. In addition, there are no established therapeutic strategies for about 5–10% of patients who have no evidence of* H. pylori* infection [7–9].

Therefore, the aim of the present study was to evaluate the long-term outcome of* H. pylori* eradication therapy for gastric MALT lymphoma according to presence of* H. pylori* infections and to identify predictive factors for unresponsiveness to eradication therapy.

## 2. Methods

### 2.1. Patient Population

A retrospective medical record review of patients who were histologically diagnosed with gastric MALT lymphoma at the Chungnam National University Hospital (Daejeon, Korea) between January 2001 and June 2014 was performed. The subjects were patients who had undergone consecutive follow-up for at least six months after treatment for gastric MALT lymphoma in this hospital; of the 63 enrolled patients, a total of 54 were analyzed after excluding 6 patients who were lost to follow-up and 3 who refused treatment. This study was approved by the Institutional Review Board of Chungnam National University Hospital (2015-04-003).

### 2.2. Diagnosis and Staging

Gastric MALT lymphoma was diagnosed according to the World Health Organization classification of lymphoid neoplasms for extranodal marginal zone B-cell lymphoma of the MALT type, and the Lugano staging system [10] was used to determine the clinical stage. Lesion shape, location, and number were analyzed based on endoscopic findings. Lesions were morphologically classified as superficial or advanced cancer-like types, and they were also classified according to their location in the stomach: proximal (cardia, fundus, upper body, and midbody) or distal (lower body, antrum, and pylorus).

### 2.3. Evaluation and Eradication of* H. pylori* Infection

*H. pylori* infection was confirmed with at least two of the following tests: histology, urea breath test, or rapid urease test (Hp Kit, Chongkundang Pharmaceutical, Republic of Korea). Patients with at least one positive test result were defined as* H. pylori*-positive, and those with all negative results were considered* H. pylori-*negative. For first-line eradication therapy a proton pump inhibitor- (PPI-) based triple therapy was administered for 1-2 weeks: PPI (standard dose b.i.d.), clarithromycin (0.5 g b.i.d.), and amoxicillin (1 g b.i.d.). Urea breath tests were performed in all patients at least 4–6 weeks after treatment completion to confirm* H. pylori* eradication. For patients with failed first-line triple therapy, a second-line eradication therapy consisting of PPI (standard dose b.i.d.), tripotassium dicitrato bismuthate (300 mg q.i.d.), metronidazole (500 mg t.i.d.), and tetracycline (500 mg q.i.d.) was administered for 1-2 weeks.

### 2.4. Response Assessment

Posttreatment response was classified into four groups based on the grading system from the European Gastro-Intestinal Lymphoma Study (EGILS) [6]. Complete remission (CR) was defined as no macroscopic findings of lymphoma and negative histologic findings in at least two subsequent follow-up investigations. Partial remission (PR) was defined as reduction of macroscopic and histologic findings. Stable disease (SD) was characterized as unmodified macroscopic and/or histologic findings. Progressive disease (PD) was defined as the worsening of macroscopic or histologic findings. Patients other than those who met the CR criteria at the follow-up of at least 6 months (*H. pylori*-negative patients) or 12 months (*H. pylori*-positive patients) after* H. pylori* eradication were defined nonresponders. In all patients, follow-up endoscopy with multiple biopsies was performed every 3–6 months after therapy completion until CR was achieved. In the case of CR, regular endoscopic surveillance was performed every 6–12 months.

### 2.5. Statistical Analysis

Each categorical variable was analyzed using chi-square or Fisher's exact tests in order to compare the baseline characteristics of* H. pylori*-positive and* H. pylori*-negative patients with gastric MALT lymphoma. The odds ratios of predictive factors of resistance to eradication therapy were analyzed with a logistic regression model. For multivariate analysis, some variables that were considered clinically significant were adjusted. All analyses were conducted using SPSS version 19.0 software (SPSS Inc., Chicago, IL, USA); *P* values were two-sided, and *P* values less than 0.05 were considered significant.

## 3. Results

### 3.1. Clinical Characteristics of* H. pylori*-Positive and* H. pylori*-Negative Groups

A total of 54 patients with gastric MALT lymphoma were enrolled in the study; of these, 42 (78%) were* H. pylori*-positive. The mean age (standard deviation, SD) was 58.8 (9.8) years (range: 38–82 years). The baseline characteristics according to* H. pylori* infection status are shown in Table 1. The lesions were located more frequently in both the proximal and distal parts of the stomach (*P* = 0.001), and the percentage of multiple lesions was significantly greater in the* H. pylori*-negative group than in the* H. pylori*-positive group (*P* = 0.046). However, other characteristics, including age, sex, endoscopic findings, and clinical stage, were not significantly different between the two groups.

### 3.2. *H. pylori* Eradication and Response to* H. pylori* Eradication

The treatment response of each group of patients is shown in Table 2. The median follow-up period was 51 months (range: 7–156 months), and all patients except two had localized gastric MALT lymphoma (stage I). Of the 42* H. pylori-*positive patients, 41 received eradication therapy. Of them, 35 patients achieved successful eradication of* H. pylori* (85%): 33 patients who received first-line triple therapy and 2 of 8 patients who received second-line quadruple eradication therapy. As a result of the eradication therapy, all of these 35 patients (100%) achieved CR. Six of 12* H. pylori*-negative patients (50%) underwent eradication therapy. Three of these six patients (50%) showed CR. The median time to reach CR after the completion of eradication therapy tended to be longer in* H. pylori*-negative patients (6.1 months) than in* H. pylori*-positive patients (4.8 months). However, it was not significantly different between the two groups.

### 3.3. Characteristics of Treatment Responders and Nonresponders

Among 47 patients who chose eradication therapy as first-line treatment, 38 (80.9%) showed a response, while 9 did not; the clinical characteristics of the groups are shown in Table 3. The proportion of patients with multiple lesions was significantly greater in the nonresponder group (8 patients, 89%) than in the responder group (16 patients, 42%) (*P* = 0.023). However, other characteristics, including age, sex, lesion site, endoscopic findings, and clinical stage, did not differ significantly between the two groups. Univariate and multivariate logistic regression analysis identified that the presence of multiple lesions was the only independent predictor of resistance to* H. pylori* eradication (*P* = 0.024) (Table 4).

### 3.4. Treatments and Outcomes in* H. pylori*-Positive and* H. pylori*-Negative Patients

**Figure 1:**
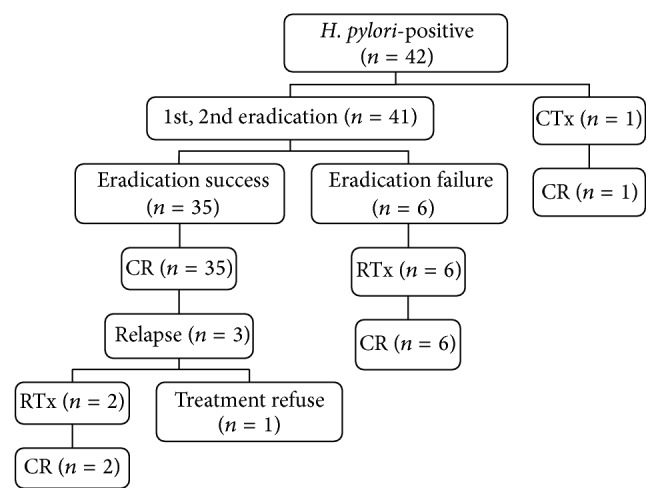
Clinical outcome of* Helicobacter pylori*-positive gastric mucosa-associated lymphoid tissue (MALT) lymphoma. CR: complete remission; RTx: radiotherapy; CTx: chemotherapy.

**Figure 2:**
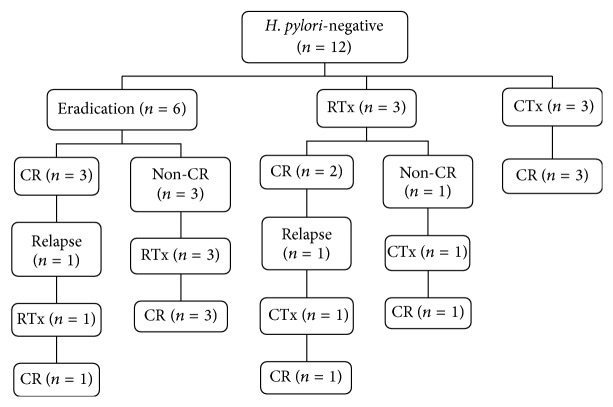
Clinical outcome of* Helicobacter pylori*-negative gastric mucosa-associated lymphoid tissue (MALT) lymphoma. CR: complete remission; RTx: radiotherapy; CTx: chemotherapy.

Posttreatment clinical outcomes in* H. pylori*-positive and* H. pylori*-negative patients with gastric MALT lymphoma are shown in Figures 1 and 2. Of 42* H. pylori*-positive patients, one patient received chemotherapy due to advanced-stage disease. Six* H. pylori*-positive patients received radiotherapy, those who did not respond to eradication therapy. Three* H. pylori*-negative patients received chemotherapy, and the remaining three patients received radiotherapy. All* H. pylori*-positive and* H. pylori*-negative patients who received chemotherapy achieved CR. All* H. pylori*-positive patients who received radiotherapy achieved CR. However, one* H. pylori*-negative patient who had no response to radiotherapy received additional chemotherapy and achieved CR. Comparison of the overall CR rate of each treatment modality was as follows: eradication therapy resulted in a CR rate of 81% (38/47), radiotherapy resulted in 93% (14/15), and chemotherapy resulted in 100% (6/6).

### 3.5. Recurrence of MALT Lymphoma

During the follow-up period, three patients in the* H. pylori*-positive group relapsed at a median of 13 months (range: 6–27 months) after remission without evidence of reinfection. Of them, one patient refused alternative treatment owing to old age, and the other two patients received radiotherapy and achieved CR. One* H. pylori*-negative patient who had undergone eradication therapy relapsed at 17 months after CR; this patient achieved CR after radiotherapy and is currently undergoing follow-up. One patient who had shown CR after radiotherapy relapsed at 13 months but achieved CR after chemotherapy. In summary, the relapse rate was 11% (4/38) after eradication therapy and 7% (1/15) after radiotherapy and there was no relapse (0/6) after chemotherapy. There was no statistical difference in the CR maintenance period or the relapse rate after CR between the* H. pylori*-positive and negative groups (Table 2).

## 4. Discussion

In the current study, 85% (35/41) of* H. pylori*-positive patients and 50% (3/6) of* H. pylori*-negative patients achieved CR after eradication. It has been demonstrated that eradication therapy has a high efficacy (approximately 80%) in* H. pylori*-positive gastric MALT lymphoma [5]. The high response rate observed in our study can be explained in part by the fact that the majority of enrolled patients (78%) were* H. pylori*-positive and that a high proportion (95%) of the patients had early-staged lesions. Our present data reconfirm the fact that* H. pylori* eradication should be the first-line treatment for localized* H. pylori*-positive gastric MALT lymphoma.

On the other hand, the efficacy of eradication therapy in* H. pylori*-negative gastric MALT lymphoma has not yet been fully determined [11]. A multicenter cohort study in Japan reported a 13.6% (6/44) response rate after eradication [12]. In addition, a systematic review with pooled analysis including 11 studies with 110 patients with early-stage* H. pylori*-negative MALT lymphoma revealed a 15.5% (17/110; 95% confidence interval, 8.7–22.2) CR rate [11]. However, many studies included in this pooled analysis enrolled a limited number of patients, and there were a wide variety of CR rates between the studies. Therefore, the effectiveness of* H. pylori* eradication therapy for* H. pylori*-negative gastric MALT lymphoma remains to be elucidated. The 2010 EGILS Group recommendations for the treatment of* H. pylori*-negative gastric MALT lymphoma state that patients “can also undergo anti-*H. pylori* treatment” [6]. The 2013 European Society of Medical Oncology guidelines recommend eradication in* H. pylori*-negative MALT lymphoma cases, albeit consideration of oncological treatments is needed [13]. Although the number of* H. pylori-*negative patients that underwent eradication therapy was small (*n* = 6) in our study, the 50% CR rate observed after eradication therapy in* H. pylori*-negative patients supports its use in this patient group. Furthermore, the present study demonstrated comparable results in terms of the median time to achieve CR after the completion of eradication therapy, the CR maintenance period, and the relapse rate between the* H. pylori*-positive and* H. pylori*-negative gastric MALT lymphoma groups. Hence, it would seem reasonable to perform eradication therapy before oncologic therapies in localized* H. pylori*-negative gastric MALT patients. Eradication therapy is expected to produce promising results not only in initial treatment responses, but also in long-term follow-up for* H. pylori*-negative MALT lymphoma patients. Further studies are necessary to confirm these results.

Research on the clinicopathologic characteristics of* H. pylori*-negative MALT lymphoma is limited and has shown inconsistent results. In the present study, no significant differences in age, sex, endoscopic findings, or clinical stage that were found in previous studies [14, 15] were observed between the two groups. Interestingly, our study showed that* H. pylori*-negative gastric MALT lymphomas more frequently involve both proximal and distal parts of the stomach (*P* = 0.001) and have a higher frequency of multiple lesions compared to the* H. pylori*-positive group (*P* = 0.046). Some studies have indicated that although gastric MALT lymphomas can occur in any region of the stomach, they are frequently located in the distal portion of the stomach, suggesting that they are associated with the localization of the highest concentration of colonized* H. pylori* organisms and acquired lymphoid tissue [7, 16, 17]. Further studies with a larger number of patients are needed to verify this difference.

Some predictive factors for resistance to* H. pylori* eradiation in gastric MALT lymphoma have been demonstrated, such as the absence of* H. pylori* infection [8, 14, 15, 18], advanced stage [8, 14], a diffuse large B-cell lymphoma component [9, 19–21], proximal location [16, 22], endoscopic nonsuperficial type [18, 19], deep invasion of lymphoma in the gastric wall [20, 23], and t(11;18)/API2-MALT1 translocation [14, 22]. Our results are in line with the earlier studies, which reported that multiple locations are associated with nonresponse to therapy [11]. Though a study with a larger sample size is needed, the presence of multiple lesions should be taken into consideration before eradication therapy in patients with gastric MALT lymphoma.

A variety of relapse rates after CR in patients who have undergone* H. pylori* eradication have been reported. In a prospective, multicenter trial from Europe comprising 120 patients, 3% (3/96) of patients relapsed within 4 months, 5 months, and 24 months, respectively, after a median follow-up of 75 months [24]. In another retrospective, multicenter study enrolling 60 patients, 30.9% (13/42) recurred within a median time of 19 months (range, 3–41 months) [25]. In our study, relapse occurred in 10.5% (4/38) of patients with a median follow-up period of 51 months. Of note, lymphoma relapse occurred within a median of 17 months, with the last relapse occurring at 27 months. According to a previous study, gastric MALT lymphoma recurrence could be observed up to 131 months after CR [11]. In this regard, long-term, careful follow-up is mandatory for all patients.

Gastric MALT lymphoma progresses slowly, and its prognosis tends to be favorable [26]. Thus, eradication therapy is often administered as an initial treatment before oncologic therapy in* H. pylori*-negative patients. The slow disease progression and indolence of* H. pylori* mean that treatment delay due to a lack of response to initial eradication therapy does not greatly affect patient prognosis. In fact, one study reported that all five* H. pylori*-negative patients who had no response to eradication therapy reached regression after radiotherapy [15], a finding similar to the current study, in which all three patients who had no response to eradication therapy achieved CR without recurrence after radiotherapy. Considerations of stomach preservation and morbidity for the treatment of localized gastric MALT lymphoma have recently diminished the role of surgery in favor of radiotherapy or chemotherapy, with satisfactory results [27, 28]. Likewise, the treatment success rate in the present study was 93% for radiotherapy and 100% for chemotherapy in all patients, regardless of their* H. pylori* infection status, and recurrence was low (radiotherapy: 7%, chemotherapy: 0%). Therefore, in patients with gastric MALT lymphoma, radiotherapy or chemotherapy should be the first choice for those who have experiences with therapy failure or in those who are predicted to have resistance to eradication therapy.

This study had several limitations. First, selection bias was possible owing to the small sample size of* H. pylori*-negative gastric MALT lymphoma patients. It was difficult to enroll many patients because of the low incidence of gastric MALT lymphoma. Second, we were unable to study the status of the t(11;18)/API2-MALT1 translocation, which is known to be an important factor for predicting eradication therapy response in patients with gastric MALT lymphoma. Third, there was a possibility that some treatment responders were categorized as nonresponders; in some patients, the evaluation of response to therapy occurred too soon after eradication therapy, and it was difficult to apply the “watch and wait” strategy to all patients.

In conclusion,* H. pylori* eradication therapy could be an effective first-line treatment option in localized* H. pylori*-negative gastric MALT lymphoma, especially for patients with single lesions. Initial treatment responses as well as long-term outcomes in* H. pylori*-negative gastric MALT lymphoma are comparable to those in* H. pylori*-negative lymphoma.

## Figures and Tables

**Table 1:** Baseline characteristics of *H. pylori*-positive and *H. pylori*-negative gastric mucosa-associated lymphoid tissue lymphoma patients.

Variables	*H. pylori*+ (*n* = 42)	*H. pylori*− (*n* = 12)	*P* value
Mean age (SD)	58.9 (11.5)	58.5 (6.7)	0.651
Gender (%)			0.051
Male	14 (33)	8 (67)	
Female	28 (67)	4 (33)	
Site of lesion (%)			0.001
Proximal^*∗*^	23 (55)	4 (33)	
Distal^†^	15 (36)	2 (17)	
Both	4 (9)	6 (50)	
Number of lesions (%)			0.046
Single	22 (52)	2 (17)	
Multiple	20 (48)	10 (83)	
Endoscopic finding (%)			0.356
Superficial type	37 (88)	9 (75)	
Advanced cancer-like type	5 (12)	3 (25)	
Clinical stage (%)			0.067
I	40 (95)	9 (75)	
II or more	2 (5)	3 (25)	

^*∗*^Cardia, fundus, high body, or midbody. ^†^Low body, angle, or antrum.

SD: standard deviation; *H. pylori*: *Helicobacter pylori*.

**Table 2:** Clinical response to eradication therapy according to *H. pylori *infection status.

Variables	*H. pylori*+ (*n* = 42)	*H. pylori*− (*n* = 12)	*P* value
Initial *H. pylori *eradication (%)	41 (100)^*∗*^	6 (50)^†^	
Successful eradication (%)	35 (85)		
Response to *H. pylori *eradication (%)			
Complete remission	35 (85)	3 (50)	0.133
Partial remission	2 (5)	0 (0)	
Stable disease	4 (10)	2 (33)	
Progressive disease	0 (0)	1 (17)	
Median time to achieve CR (month, SD)	4.8 (1.1)	6.1 (1.5)	0.108
CR maintenance period (month, SD)	28.0 (19.1)	32.0 (18.0)	0.442
Relapse rate after CR	3 (9)	1 (33)	0.718

^*∗*^One patient received chemotherapy initially due to advanced stage. ^†^Three patients underwent chemotherapy and other three patients were treated with radiotherapy initially.

SD: standard deviation; *H. pylori*: *Helicobacter pylori;* CR: complete remission.

**Table 3:** Clinical characteristics of responder and nonresponder group after eradication therapy.

Variables	Responder (*n* = 38)	Nonresponder (*n* = 9)	*P* value
Mean age (SD)	58.7 (10.8)	59.2 (11.4)	0.259
Gender (%)			0.252
Male	12 (32)	5 (56)	
Female	26 (68)	4 (44)	
*H. pylori* status (%)			0.075
Positive	35 (92)	6 (67)	
Negative	3 (8)	3 (33)	
Site of lesion (%)			0.270
Proximal^*∗*^	23 (61)	3 (33)	
Distal^†^	11 (29)	5 (56)	
Both	4 (10)	1 (11)	
Number of lesions (%)			0.023
Single	22 (58)	1 (11)	
Multiple	16 (42)	8 (89)	
Endoscopic finding (%)			0.302
Superficial type	32 (84)	8 (89)	
Advanced cancer-like type	6 (16)	1 (11)	
Clinical stage (%)			0.650
I	36 (95)	9 (100)	
II or more	2 (5)	0 (0)	

^*∗*^Cardia, fundus, high body, or midbody. ^†^Low body, angle, or antrum.

SD: standard deviation; *H. pylori*: *Helicobacter pylori*.

**Table 4:** Predictive factor for resistance to *H. pylori *eradication by logistic regression analysis.

Variables	Univariate analysis		Multivariate analysis	
	OR (95% CI)	*P* value	OR (95% CI)	*P* value
Age (elderly)	1.12 (0.81–1.45)	0.289		
Gender (male)	1.26 (0.69–1.83)	0.325		
*H. pylori* status (negative)	5.83 (0.95–35.10)	0.057	4.71 (0.89–31.58)	0.073
Number of lesions (multiple)	8.22 (1.21–51.48)	0.019	7.83 (1.16–49.81)	0.024
Location of lesion (proximal)	0.29 (0.06–1.42)	0.127		

*H. pylori*: *Helicobacter pylori*; OR: odds ratio; CI: confidence interval.
